# The permeability of human red blood cell membranes to hydrogen peroxide is independent of aquaporins

**DOI:** 10.1016/j.jbc.2021.101503

**Published:** 2021-12-18

**Authors:** Florencia Orrico, Ana C. Lopez, Daniela Saliwonczyk, Cecilia Acosta, Ismael Rodriguez-Grecco, Isabelle Mouro-Chanteloup, Mariano A. Ostuni, Ana Denicola, Leonor Thomson, Matias N. Möller

**Affiliations:** 1 Laboratorio de Fisicoquímica Biológica, Instituto de Química Biológica, Facultad de Ciencias, Universidad de la República, Montevideo, Uruguay; 2 Laboratorio de Enzimología, Instituto de Química Biológica, Facultad de Ciencias, Universidad de la República, Montevideo, Uruguay; 3 Centro de Investigaciones Biomédicas (CEINBIO), Universidad de la República, Montevideo, Uruguay; 4 Departamento de Medicina Transfusional, Hospital de Clínicas, Facultad de Medicina, Universidad de la República, Montevideo, Uruguay; 5 UMR_S1134, BIGR, Inserm, Université de Paris, Paris, France; 6 Laboratoire d'Excellence GR-Ex, Paris, France

**Keywords:** hydrogen peroxide, permeability, membrane, liposome, red blood cell, erythrocyte, diffusion, catalase, aquaporin, AQP1, aquaporin 1, AQP3, aquaporin 3, AQP8, aquaporin 8, Chol, cholesterol, DMPC, dimiristoylphosphatidylcholine, DOPC, dioleoylphosphatidylcholine, DPPG, dipalmitoylphosphatidylglycerol, HbO_2_, oxyhemoglobin, H_2_O_2_, hydrogen peroxide, pCMBS, *p*-chloromercuribenzenesulfonic acid, POPG, phosphatidylglycerol, Prx2, peroxiredoxin 2, RBC, red blood cell, USL, unstirred layer

## Abstract

Hydrogen peroxide (H_2_O_2_) not only is an oxidant but also is an important signaling molecule in vascular biology, mediating several physiological functions. Red blood cells (RBCs) have been proposed to be the primary sink of H_2_O_2_ in the vasculature because they are the main cellular component of blood with a robust antioxidant defense and a high membrane permeability. However, the exact permeability of human RBC to H_2_O_2_ is neither known nor is it known if the mechanism of permeation involves the lipid fraction or protein channels. To gain insight into the permeability process, we measured the partition constant of H_2_O_2_ between water and octanol or hexadecane using a novel double-partition method. Our results indicated that there is a large thermodynamic barrier to H_2_O_2_ permeation. The permeability coefficient of H_2_O_2_ through phospholipid membranes containing cholesterol with saturated or unsaturated acyl chains was determined to be 4 × 10^−4^ and 5 × 10^−3^ cm s^−1^, respectively, at 37 °C. The permeability coefficient of human RBC membranes to H_2_O_2_ at 37 °C, on the other hand, was 1.6 × 10^−3^ cm s^−1^. Different aquaporin-1 and aquaporin-3 inhibitors proved to have no effect on the permeation of H_2_O_2_. Moreover, human RBCs devoid of either aquaporin-1 or aquaporin-3 were equally permeable to H_2_O_2_ as normal human RBCs. Therefore, these results indicate that H_2_O_2_ does not diffuse into RBCs through aquaporins but rather through the lipid fraction or a still unidentified membrane protein.

Hydrogen peroxide (H_2_O_2_) is probably one of the most abundant reactive species derived from oxygen in biology. It can be produced enzymatically by several oxidases and also as a byproduct of mitochondrial respiration (1). Cells are equipped to deal with excess H_2_O_2_ with several enzymatic antioxidant defenses, including peroxiredoxins, glutathione peroxidases, and catalases. H_2_O_2_-triggered signaling has been associated to various physiological responses, such as cell migration, growth, and proliferation (1). It has been proposed that the signaling is mediated by a redox relay system, involving the oxidation of a peroxiredoxin that then conveys the oxidation equivalents to a second protein, likely with the assistance of an adapter protein (2, 3).

In the vasculature, H_2_O_2_ is produced mainly by NADPH oxidases from endothelial cells. Once formed, it can diffuse and cause vasodilation (4, 5) or promote cell growth, proliferation, and migration in endothelial and smooth muscle cells (6, 7). There have been several attempts to measure the steady-state concentration of H_2_O_2_ in blood. A normal range of 1 to 5 μM, which could increase up to 50 μM under inflammatory conditions, has been estimated (8). However, the great variations in the reported values indicate that it is a challenging task. Very likely, the actual concentration of H_2_O_2_ is much lower because red blood cells (RBCs) consume H_2_O_2_ rapidly and efficiently. In fact, RBCs contain a robust antioxidant defense consisting of peroxiredoxin 2 (Prx2), glutathione peroxidase 1, and catalase (9). Physiological low amounts of H_2_O_2_, including those produced by oxyhemoglobin (HbO_2_) autoxidation, are consumed mainly by Prx2, which is very abundant (240–520 μM) (10, 11) and reacts very rapidly (*k* = 1 × 10^8^ M^−1^s^−1^, (12)). The reduction of Prx2 back to the active state by thioredoxin and thioredoxin reductase is limited by the low amount of NADPH present in the RBC, so large amounts of H_2_O_2_ can transiently inactivate Prx2 (9, 13). In such a scenario, catalase consumes most of the remaining H_2_O_2_, at a slower rate (9).

H_2_O_2_ is consumed by enzymes that are located in the cytosol of RBCs; therefore, it can cross the plasma membrane. Notably, the permeability of human RBC membrane to H_2_O_2_ has not been determined but has been estimated in a few studies to be 7 × 10^−4^ and 1.1 × 10^−3^ cm s^−1^ (9, 14), respectively. The permeability of RBCs from rat and horse has been determined to be 1.2 × 10^−2^ cm s^−1^ and 6 × 10^−4^ cm s^−1^, respectively (15, 16), indicating a significant variability and prompting the determination of the permeability of human RBCs to H_2_O_2_. The permeability of several cells to H_2_O_2_ has been quantitatively determined and found to range from 2 × 10^−4^ cm s^−1^ in Jurkat T cells to 1.6 × 10^−3^ cm s^−1^ in human umbilical vein endothelial cells (17).

The role of aquaporins (AQPs) in facilitating the transport of H_2_O_2_ has been studied qualitatively in different systems. It was found that not all AQPs, but certain isoforms later termed peroxiporins, facilitate the transport of H_2_O_2_ (18). Among these are human aquaporin 3 (AQP3) and human aquaporin 8 (AQP8) (18, 19, 20, 21, 22). Human aquaporin 1 (AQP1), on the contrary, did not appear to facilitate H_2_O_2_ transport (18, 22), but this has recently been disputed and remains controversial (23, 24). AQP1 is one of the most abundant proteins in the membrane of RBCs, and AQP3 is also present at lower levels (11), so H_2_O_2_ may diffuse across the RBC membrane through these channels.

In contrast to the information on permeability of cellular membranes to H_2_O_2_, little is known about the permeability of lipid-only membranes that would mimic the lipid fraction of cell membranes. In these membranes, permeation would occur by simple diffusion, a process known to be greatly affected by solubility of the permeant molecule in the lipid phase (25, 26). Some reports have shown changes in diffusion rates depending on liposome composition, temperature, and compressibility, but no permeability coefficient (*P*_m_) values have been determined (15, 27, 28). Molecular dynamics simulations have provided detailed information on H_2_O_2_ distribution across the lipid bilayer, but with no experimental counterpart to validate it (29, 30, 31). This lack of information somehow leads to the idea that H_2_O_2_ can only traverse cell membranes by facilitated transport through AQPs, disregarding simple diffusion.

Herein, we first determined the solubility of H_2_O_2_ in organic solvents representing different depths in the membrane, to gain insight of the thermodynamic barrier to H_2_O_2_ diffusion across lipid membranes. To measure the *P*_m_ of lipid membranes, we used liposomes made of cholesterol (Chol) and phospholipids containing saturated or unsaturated acyl chains, which encapsulated catalase. The *P*_m_ of these membranes to H_2_O_2_ was determined by comparing the rate of decomposition of H_2_O_2_ by intact *versus* disrupted liposomes, using the enzyme latency principle. The same approach was used to determine the *P*_m_ of human RBCs to H_2_O_2_. Finally, we studied the permeation mechanism of H_2_O_2_ in human RBCs, evaluating in the process the potential role of AQPs in H_2_O_2_ transport.

## Results

### Solubility of H_2_O_2_ in organic solvents

As a first approximation to the solubility of H_2_O_2_ in the lipid membrane, we used organic solvents that have been previously used to mimic several physicochemical properties of lipid membranes, namely *n*-octanol and hexadecane (25, 32, 33). As expected, the solubility of H_2_O_2_ in organic solvents was significantly lower than in water. The solubility in *n*-octanol was 15 times lower than in water, whereas in hexadecane, 122,000 times lower than in water (Table 1). Temperature-dependent assays further indicate that there is a greater contribution of enthalpy than entropy to the transfer of H_2_O_2_ from water to these solvents, consistent with an important loss of hydrogen bonds of H_2_O_2_ on transferring from water to the organic phase (Table 1).

**Table 1:** Partition constants of H_2_O_2_ between organic solvents and water at 25 °C

Organic solvent	Dielectric constant	*K*_*D*_°	Δ*G*° (kJ mol^−1^)	Δ*H*° (kJ mol^−1^)	Δ*S*° (J mol^−1^ K^−1^)
Octanol	10.3	(6.6 ± 0.4) × 10^−2^	6.7	5.9	−2.6
Hexadecane	2.05	(8.2 ± 0.6) × 10^−6^	29.0	26.4	−8.7

For comparison, the dielectric constant of water is 78.4.

**Figure 1:**
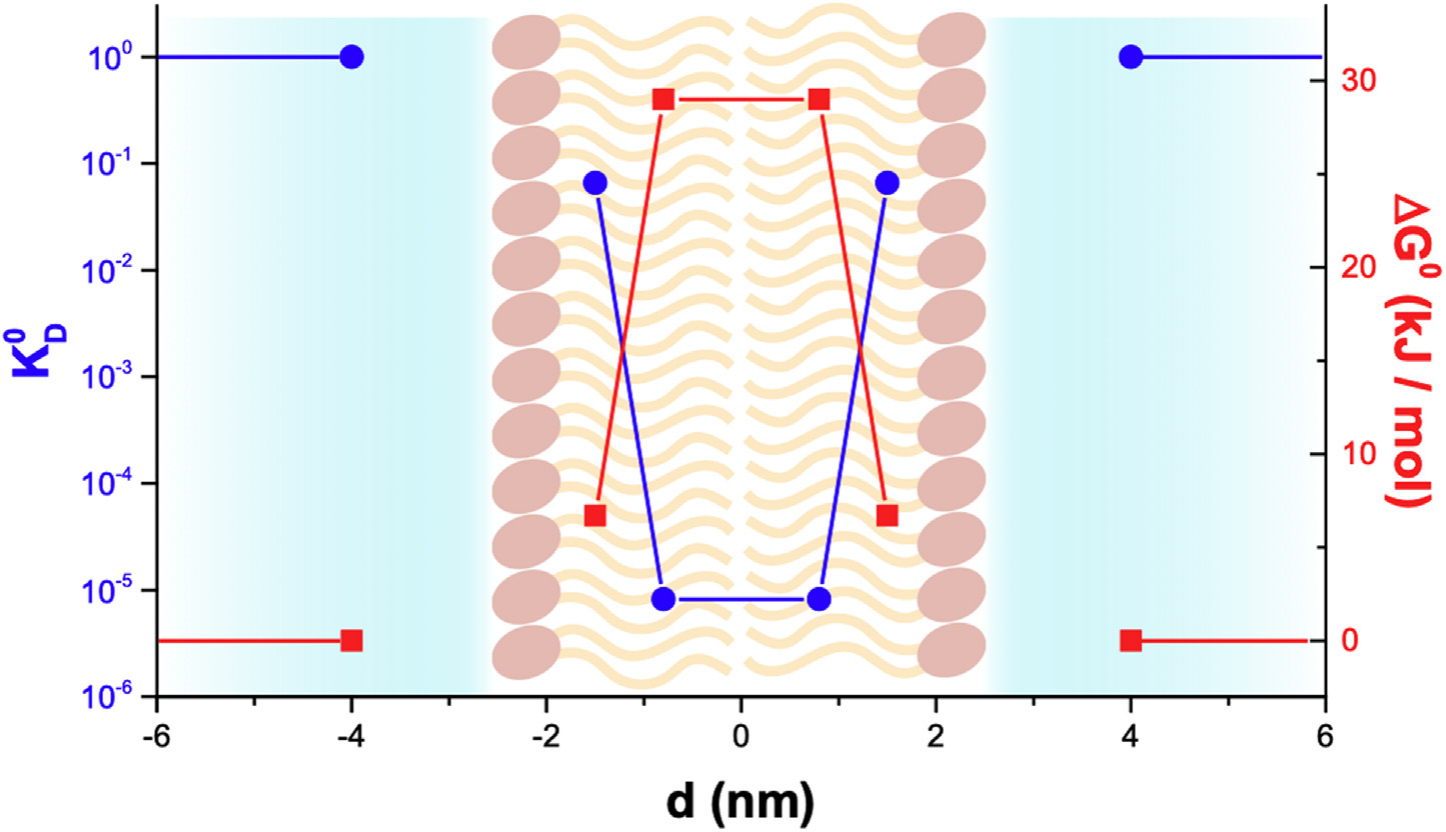
**Estimated solubility profile of H**_**2**_**O**_**2**_**across the lipid bilayer, according to *K***_***D***_**° (*blue*) and Δ*G*° (*red*).** Assuming a uniform diffusion coefficient of H_2_O_2_ across the membrane (*D* = 1.4 × 10^−5^ cm^2^ s^−1^, (39)), and using Equation 1, a *P*_m_ = 7 × 10^−4^ cm s^−1^ is estimated. H_2_O_2_, hydrogen peroxide.

Hexadecane and *n*-octanol differ in polarity as evidenced by the different dielectric constants (Table 1) and can be considered to represent different regions of the membrane (34, 35, 36). The relatively more polar *n*-octanol is similar to the acyl region near the carbonyl groups, whereas the less polar hexadecane is similar to the middle-bilayer region, thus a solubility profile across the membrane can be estimated. Figure 1 shows the estimated solubility profile across the membrane as well as the Gibbs energy of partition (Δ*G*°) associated to such position. The profile is discontinuous between the membrane and bulk water because we have no experimental estimation on the solubility of H_2_O_2_ in the highly polar headgroup region or the structured water region near the headgroups.

The *P*_m_ can be calculated in an inhomogeneous media such as the membrane as the inverse of the sum of resistances to permeation at the different depths through the membrane (37, 38):

(1) $$P_{\text{m}}={\left(\overset{\text{d}/2}{\underset{-\text{d}/2}{\int }}\frac{1}{K\left(\text{z}\right)D\left(\text{z}\right)}\text{dz}\right)}^{-1}$$

where *K*(z) and *D*(z) are the *K*_*D*_° and diffusion coefficient at a depth z in the membrane. To estimate *P*_m_, it was assumed that *D* for H_2_O_2_ is the same across all the membrane and equal to that in water (*D* = 1.4 × 10^−5^ cm^2^ s^−1^, (39)). Two regions with different solubility were considered, the central region, 1.6 nm wide, where *K*_*D*_° is given by hexadecane, and two equal regions on either side, 0.7 nm wide each, where *K*_D_° is given by octanol. The headgroup region or the structured water region adjacent to the headgroups was not considered. The estimated *P*_m_ is 7 × 10^−4^ cm s^−1^. Of course, this is a simplistic estimation that does not take into account properties of lipid bilayers such as lipid packing and composition, and so experimental determinations of *P*_m_ were done next.

### Permeability of lipid membranes to H_2_O_2_

**Figure 2:**
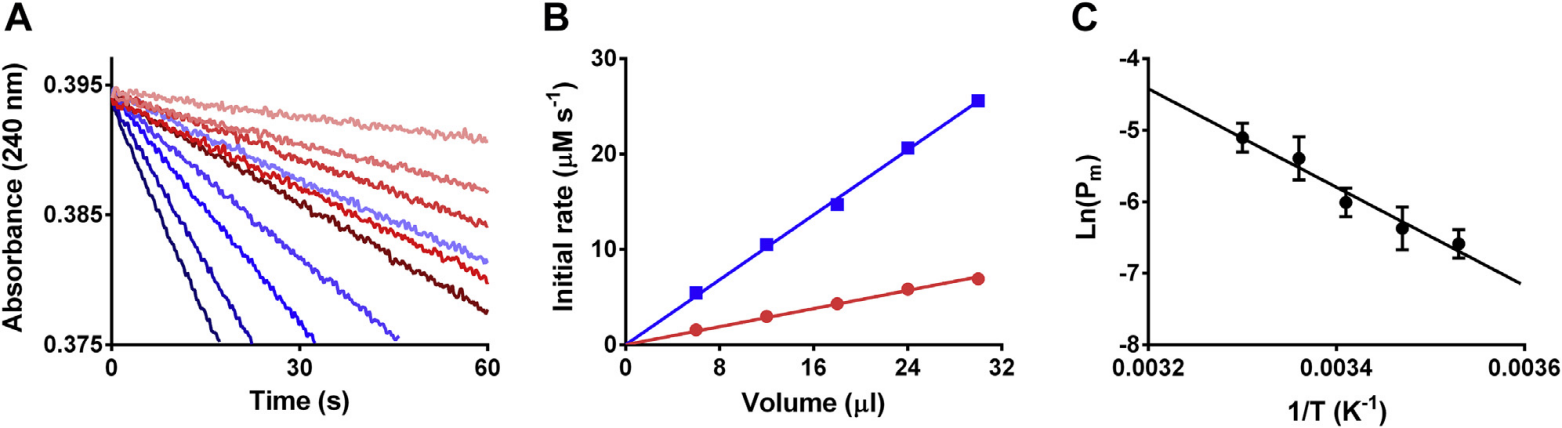
**Permeability of H**_**2**_**O**_**2**_**through DMPC:DPPG:Chol (4:1:5) membranes.***A*, initial rate determination of 10 mM H_2_O_2_ consumption by liposome-encapsulated catalase performed with increasing volumes of liposomes (5–25 μl, *red tones*) and released catalase in disrupted liposomes (5–25 μl, *blue tones*) at 25 °C. *B*, the initial rates are used to obtain pseudo–first-order constants for H_2_O_2_ consumption, *k*_lipo_ for intact (*red circles*) and *k*_dis_ for disrupted (*blue squares*) liposomes. The ratio *k*_lipo_/*k*_dis_ yields *R*_H2O2_, which is used to calculate *P*_m_ according to Equation 2. *C*, activation energy of H_2_O_2_ permeation determined in the 20 to 40 °C range, *E*_a_ = 57 ± 8 kJ mol^−1^ (n = 3). Chol, cholesterol; DMPC, dimiristoylphosphatidylcholine; DPPG, dipalmitoylphosphatidylglycerol; H_2_O_2_, hydrogen peroxide.

The permeability to H_2_O_2_ was first measured in membranes containing dimiristoylphosphatidylcholine (DMPC) (0.4 mole fraction), as the base phospholipid, dipalmitoylphosphatidylglycerol (DPPG) (0.1 mole fraction), to provide a net negative charge to the vesicles and prevent them from fusing (40), and Chol (0.5 mole fraction), to provide mechanical stability and mimic typical mammal membrane composition. It was found that catalase encapsulated in liposomes caused H_2_O_2_ decomposition in a dose-dependent manner, where the slope is the pseudo–first-order rate constant *k*_lipo_ (Fig. 2). The disruption of these liposomes by repeated extrusion through 30 nm pore filters resulted in an increase in the rate of H_2_O_2_ decomposition, consistent with release of encapsulated catalase, re-equilibration between external and internal volumes, and the loss of the permeability barrier. The rate of H_2_O_2_ decomposition was also dose dependent and used to calculate *k*_dis_ (Fig. 2). The ratio between *k*_lipo_ and *k*_dis_ yields *R*_H2O2_.

The *P*_m_ was determined based on the enzyme latency method (16, 41, 42). Briefly, it is calculated considering that at steady state, the rate of H_2_O_2_ diffusion through the membrane into the liposome will be equal to the sum of the rates of H_2_O_2_ diffusion out of the liposomes and consumption by catalase inside the liposome (41). The advantage of the latency method is that the rate of the enzymatic reaction does not have to be much higher than the diffusion rate across the membrane but only slightly higher or in the order. It is considered that H_2_O_2_ can diffuse into the vesicle and react with catalase and part of it can diffuse back to the external solution. A steady state is achieved rapidly, and then a competition between catalase decomposition and diffusion ensues, which generates a concentration gradient of H_2_O_2_ across the membrane. The gradient of concentration of H_2_O_2_ formed across the membrane is the inverse of *R*_H2O2_.

A detailed explanation of the enzyme latency method is given in the Supporting Information. The final equation used to calculate *P*_m_ is given in Equation 2.

(2) $$P_{\text{m}}=\frac{k_{catalase\;}{\text{R}}_{\text{H}2\text{O}2}}{\frac{\text{A}}{\text{V}}\left(1-{\text{R}}_{\text{H}2\text{O}2}\right)}$$

where A/V is the surface area to internal volume ratio, calculated from the hydrodynamic radii (A/V ≈ 3/r, disregarding the contribution of water to the hydrodynamic radius of these liposomes, calculated to be less than 4%). The rate constant *k*_*catalase*_ is the pseudo–first-order rate constant of catalase inside the liposome and was determined from the catalase work solution used to prepare the liposomes. It was assumed that the concentration of catalase inside the liposomes was the same as in the catalase work solution. It was a reasonable assumption because the liposomes were prepared and used the same day, and they were found to be stable (same catalase activity) for at least 3 days. Furthermore, the resulting *P*_m_ values were found to be consistent between batches from different preparations.

At 25 °C, it was found that *P*_m_ = (3.7 ± 0.5) × 10^−5^ cm s^−1^ for liposomes made of DMPC, DPPG, and Chol (DMPC:DPPG:Chol, 4:1:5 mole fraction), whereas this value increased to (1.3 ± 0.7) × 10^−3^ cm s^−1^ for liposomes made of dioleoylphosphatidylcholine (DOPC), 1-palmitoyl-2-oleoyl-phosphatidylglycerol, and Chol (DOPC:POPG:Chol, 4:1:5 mole fraction), pointing that the permeability to H_2_O_2_ depended on the degree of unsaturation of the membrane lipids. The permeability increased with temperature. At 37 °C, *P*_m_ = (4.1 ± 0.5) × 10^−4^ and (5.5 ± 0.3) × 10^−3^ cm s^−1^ for DMPC:DPPG:Chol (4:1:5) and DOPC:POPG:Chol (4:1:5), respectively (Table 2). The Arrhenius activation energy (*E*_a_) of the permeation process was calculated for each membrane and found to be 130 ± 20 and 57 ± 8 kJ mol^−1^ for DMPC:DPPG:Chol (4:1:5) and DOPC:POPG:Chol (4:1:5), respectively (Table 2).

**Table 2:** Permeability coefficients and activation energies of permeation for H_2_O_2_ in the different membranes

Liposome or cellular membrane	*P*_m_ (cm s^−1^)		*E*_a_ (kJ mol^−1^)
	25 °C	37 °C	
DMPC:DPPG:Chol (4:1:5 mole fraction)	(3.7 ± 0.5) × 10^−5^	(4.1 ± 0.5) × 10^−4^	130 ± 20
DOPC:POPG:Chol (4:1:5 mole fraction)	(1.3 ± 0.7) × 10^−3^	(5.5 ± 0.3) × 10^−3^	57 ± 8
Intact RBC	(1.0 ± 0.2) × 10^−3^	(1.6 ± 0.3) × 10^−3^	32 ± 4

*P*_m_ is reported as the mean ± the standard deviation of at least three independent determinations.

The permeability of membranes to H_2_O_2_ greatly depended on lipid composition. Membranes composed of DOPC:POPG:Chol (4:1:5) were 1 to 2 orders of magnitude more permeable to H_2_O_2_ than membranes composed of DMPC:DPPG:Chol (4:1:5), suggesting that unsaturations in the acyl chain greatly favored H_2_O_2_ partition and diffusion across the membrane. Thus, lipid membranes show different permeability to H_2_O_2_, depending on the composition, but what happens in the more complex intact RBC membrane?

### Permeability of human RBC membrane to H_2_O_2_

**Figure 3:**
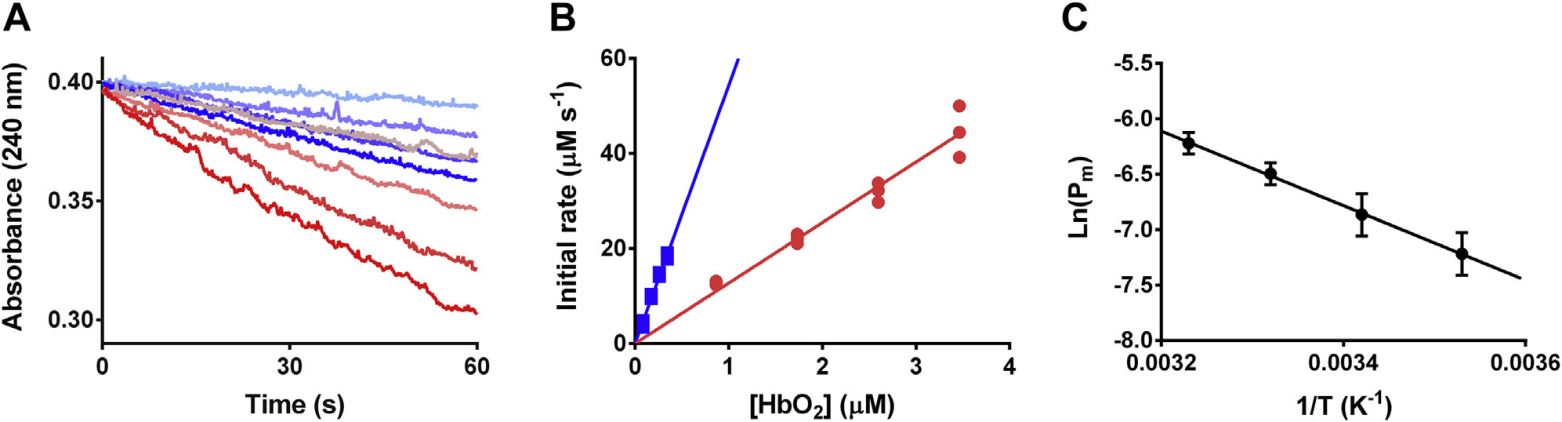
**Determination of *P***_**m**_**of RBC membranes to H**_**2**_**O**_**2**_**.***A*, consumption of 10 mM H_2_O_2_ by intact RBCs at increasing cell densities (0.9–3.5 μM HbO_2_, *red tones*) and by lysed RBCs (90–350 nM HbO_2_, *blue tones*) at 37 °C. *B*, initial rates of H_2_O_2_ consumption as a function of HbO_2_ concentration for intact (*red circles*) and lysed (*blue squares*) cells. The slopes are the respective observed rate constants *k*_RBC_ and *k*_lys_. The ratio between *k*_RBC_ and *k*_lys_ was used to calculate *R*_H2O2_ and determine *P*_m_ according to Equation 2 (n = 3, three biological replicates). *C*, estimation of the *E*_a_ of the H_2_O_2_ permeation process in RBCs, *E*_a_ = 32 ± 4 kJ mol^−1^ (n = 3). H_2_O_2_, hydrogen peroxide; HbO_2_, oxyhemoglobin; RBC, red blood cell.

The enzyme latency assay was also used to determine the *P*_m_ to H_2_O_2_ in human RBCs. To comply with the requirements of the technique, the experiments were performed with very low cell densities, using the HbO_2_ concentration in the sample as a reference, and with high H_2_O_2_ concentrations (10 mM). Given these conditions, catalase is the main enzyme involved in H_2_O_2_ decomposition (Fig. S1), as it was demonstrated previously (9). As shown in Figure 3, the rate of H_2_O_2_ decomposition is higher for lysed RBCs than for intact RBCs. The observed rate constant for the disappearance of H_2_O_2_ in lysed RBCs was 4.3 times greater than in intact RBCs, showing that as observed with liposomes, the RBC membrane imposes a barrier to H_2_O_2_ delaying its diffusion and decomposition by the intracellular catalase.

The results obtained with this method led to a *P*_m_ of (1.6 ± 0.3) × 10^−3^ cm s^−1^ at 37 °C. Similar experiments performed using 50 μM H_2_O_2_, drawing aliquots in time and quantifying with horseradish peroxidase and *p*-hydroxyphenylacetic acid, yielded very similar *P*_m_ (Fig. S2), validating the experiments performed with 10 mM H_2_O_2_. To consider the potential contribution of the unstirred layer (USL), which often confounds permeability measurements, especially in larger vesicles such as RBCs (38), we calculated the permeability of an USL 4 μm thick, considering *D*_H2O2_ = 1.43 × 10^−5^ cm^2^ s^−1^ (25 °C) (39). The permeability of this layer was calculated to be *P*_USL_ = 3.6 × 10^−2^ cm s^−1^, which is 36 times greater than *P*_m_ for RBCs at the same temperature ((1.0 ± 0.2) × 10^−3^ cm s^−1^). Therefore, USL effects were considered to be negligible in these measurements.

The *P*_m_ was also determined at increasing temperatures, up to 40 °C, allowing the estimation of the *E*_a_ of the H_2_O_2_ permeation process (Fig. 3). The calculated value, (32 ± 4) kJ mol^−1^, is four times lower than the *E*_a_ associated with simple diffusion through lipid membranes composed of DMPC:DPPG:Chol (4:1:5) and 1.8 times lower than DOPC:POPG:Chol (4:1:5) membranes (Table 2). Although it was expected that the lower *E*_a_ for RBCs would be associated with a higher *P*_m_ (38), this was not observed. Comparison between the liposomes and the RBC membrane can be complicated by the fact that membrane composition is different. In RBC, sphingomyelin that accounts for 25% of the total phospholipids and 14% of the total lipids may alter the behavior of the membrane. Another important difference in the lipids is the asymmetry between inner and outer leaflets (43). Probably the most important factor to explain the difference is the presence of proteins that account for 49% of the total membrane mass and may affect lipid fluidity and packing or offer alternative pathways for H_2_O_2_ diffusion. Altogether, these results are not conclusive about the importance of simple diffusion of H_2_O_2_ across RBC lipid membranes, implying that protein channels may be involved in the transport of H_2_O_2_ across the human RBC membrane.

### Role of AQPs in H_2_O_2_ membrane diffusion in human RBCs

**Figure 4:**
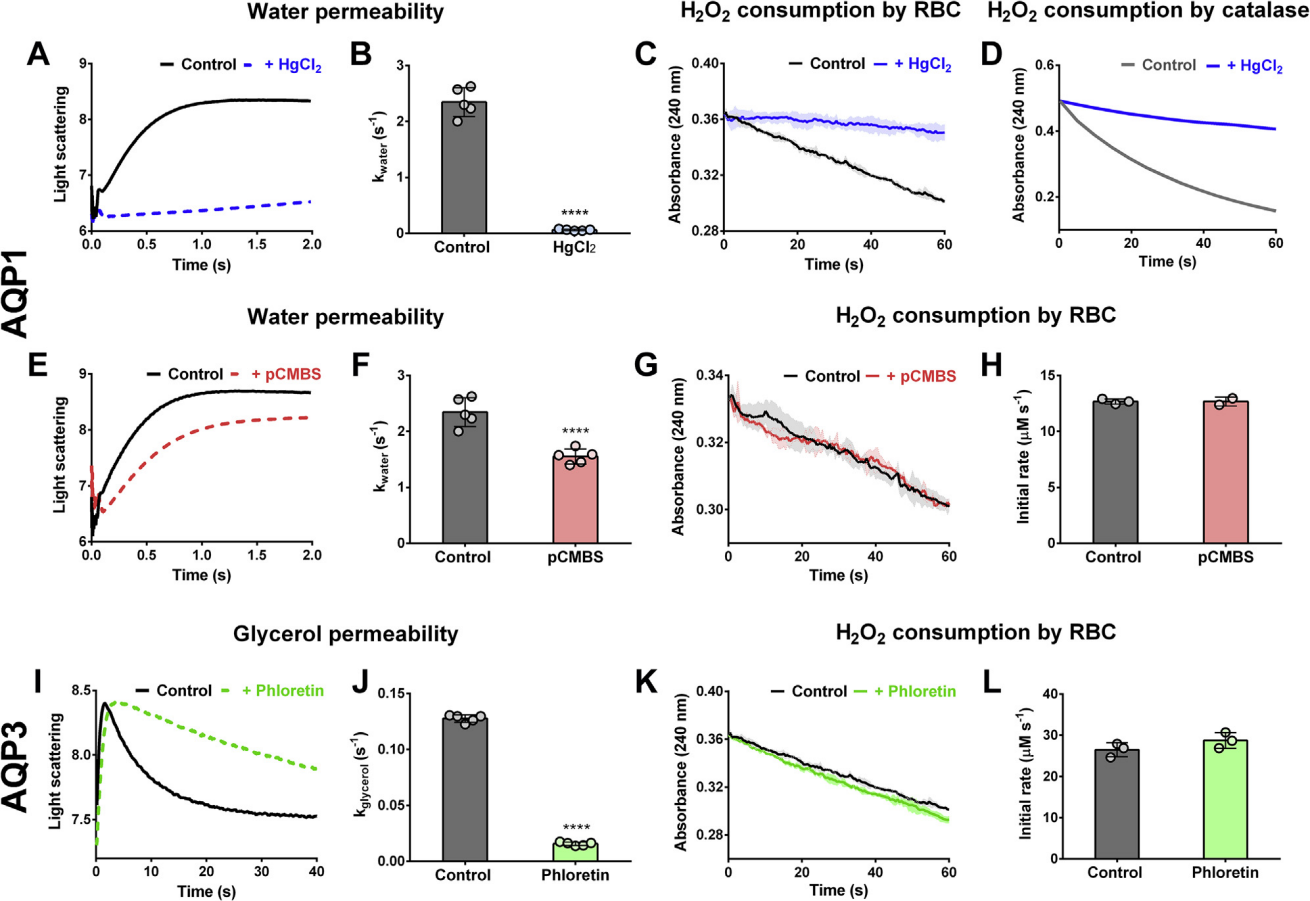
**Effect of aquaporin (AQP) inhibitors on H**_**2**_**O**_**2**_**consumption by RBCs.***A* and *E*, water efflux across the RBC membrane (0.5% hematocrit) when mixed with 250 mM sucrose. Changes in cell volume corresponding to control RBCs (*black*) and RBCs treated with 0.5 mM HgCl_2_ (*blue*) or 0.5 mM pCMBS (*red*), in order to inhibit AQP1, were monitored by light scattering measurements. *B* and *F*, observed constants (*k*_water_) obtained from the exponential fits of time courses resulting from experiments shown, and performed as, in *A* and *E*. *C*, *G*, and *K*, consumption of 10 mM H_2_O_2_ by 0.5% hematocrit suspensions of control RBCs (*black*) and RBCs treated with 0.5 mM HgCl_2_ (*blue*), 0.5 mM pCMBS (*red*) or 0.5 mM phloretin (*green*), an AQP3 inhibitor. *D*, consumption of 10 mM H_2_O_2_ by solutions of 8 nM catalase untreated (*gray*) or incubated with 0.3 mM HgCl_2_ (*blue*). *H* and *L*, initial rates of H_2_O_2_ metabolization determined from experiments shown and performed as in *G* and *K*. *I*, glycerol influx across the RBC membrane (0.5% hematocrit) followed by light scattering measurements. Untreated RBCs (*black*) or RBCs treated with 0.5 mM phloretin (*green*) were mixed with equal volumes of a 100 mM glycerol solution. *J*, observed constants (*k*_glycerol_) obtained from the nonlinear curve fit of results from experiments performed as in *I*, corresponding to the decaying phase of the time courses (∗∗∗∗*p* < 0.0001). H_2_O_2_, hydrogen peroxide; pCMBS, *p*-chloromercuribenzenesulfonic acid; RBC, red blood cell.

In other cell types, several AQP isoforms were found to facilitate H_2_O_2_ diffusion through cellular membranes (22). So studies were carried out to assess the role of AQPs in H_2_O_2_ permeability in human RBC. A series of AQP inhibitors were tested, including HgCl_2_ and *p*-chloromercuribenzenesulfonic acid (pCMBS) for AQP1 and phloretin for AQP3, incubating RBCs with each compound to evaluate possible changes in H_2_O_2_ consumption rates. The canonic (but not specific) AQP1 inhibitor HgCl_2_ resulted in complete inhibition of H_2_O_2_ decomposition by RBCs, but control experiments showed that this was caused by the inhibition of catalase, that is central to our assay (Fig. 4, *A*–*D*), and therefore could not be used. As an alternative, pCMBS was used, and even though it inhibited the transport of water through the RBC membrane as expected, it had no effect on the rate of H_2_O_2_ consumption by intact RBCs (Fig. 4, *E*–*H*). Phloretin inhibited the transport of glycerol, which occurs mostly through AQP3, but had no effect on the rate of H_2_O_2_ decomposition by intact RBCs (Fig. 4, *I*–*L*). Considering that the canonical inhibitor of water transport by AQP1, HgCl_2_, could not be used because of its effect on catalase, and that phloretin may block glycerol transport by AQP3 but not the transport of smaller molecules such as H_2_O_2_, additional assays were devised to evaluate the importance of these channels in the transport of H_2_O_2_ through the membrane of human RBCs.

To assess the participation of these water channels on the permeability to H_2_O_2_, human RBCs deficient in AQP1 and AQP3 (Colton-null and GIL-null phenotypes, respectively) were used. These samples have been extensively characterized and found that the Colton-null shows a significantly lower permeability to water, whereas the GIL-null shows normal water permeability but significantly lower permeability to glycerol (44, 45, 46). The *P*_m_ to H_2_O_2_ was determined as described previously and compared with wildtype RBCs that had been cryopreserved in the same manner as a control.

**Figure 5:**
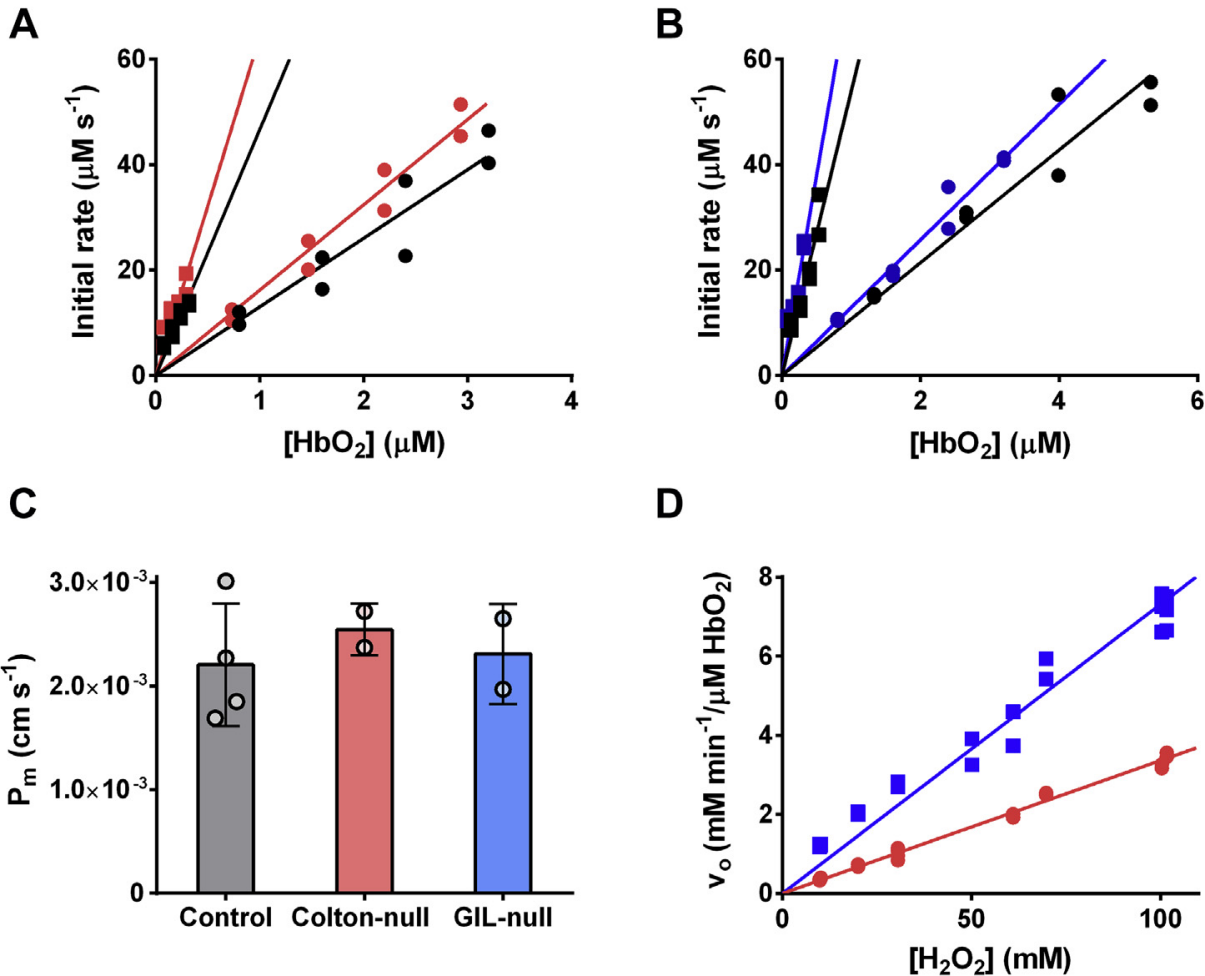
**H**_**2**_**O**_**2**_**permeability in control RBCs and RBCs lacking AQP1 (Colton-null) and AQP3 (GIL-null).***A*, secondary plot for the *P*_m_(H_2_O_2_) determination, in control (*black*) and AQP1-deficient (*red*) human RBCs. *Circles* show H_2_O_2_ consumption by intact cells, and *squares* show consumption by lysed cells. *B*, secondary plot for the *P*_m_(H_2_O_2_) determination, in control (*black*) and AQP3-deficient human RBCs (*blue*). *Circles* correspond to intact cells, and *squares* correspond to lysed cells. *C*, comparison of *P*_m_(H_2_O_2_) between control RBCs and RBCs lacking AQP1 (Colton-null) and AQP3 (GIL-null). No statistically significant differences were observed. *D*, intact (*red*) and lysed fresh RBCs (*blue*) were incubated with increasing concentrations of H_2_O_2_, from 10 to 100 mM, in HBSS solution. The initial rates were determined by stopped flow measuring absorbance at 240 nm for concentrations below 30 mM H_2_O_2_ and 270 nm for concentrations above 50 mM H_2_O_2_ (ε(H_2_O_2_) = 7.4 M^−1^ cm^−1^). No saturation of H_2_O_2_ transport or consumption by RBCs was observed. AQP1, aquaporin 1; AQP3, aquaporin 3; H_2_O_2_, hydrogen peroxide; HBSS, Hank's balanced salt solution; RBC, red blood cell.

As shown in Figure 5, cryopreserved RBC samples retained the permeability barrier to H_2_O_2_, shown by the higher *k*_lys_ (slope given by *square symbols*) compared with *k*_RBC_ (slope given by *round symbols*). The *P*_m_ for the cryopreserved control RBCs was slightly higher than *P*_m_ determined in fresh RBCs, but within experimental error. Remarkably, no differences were observed in H_2_O_2_ permeability between control, Colton-null, and GIL-null RBCs. Furthermore, using fresh RBCs, no saturation was observed in H_2_O_2_ consumption rates up to 100 mM H_2_O_2_ (Fig. 5*D*). Altogether, these results strongly suggest that neither AQP1 nor AQP3 are involved in H_2_O_2_ transport across the human RBC membrane and support that H_2_O_2_ traverses the human RBC membrane by simple diffusion across the lipid fraction or through a still unidentified protein channel.

### Physiological implications of H_2_O_2_ diffusion

**Figure 6:**
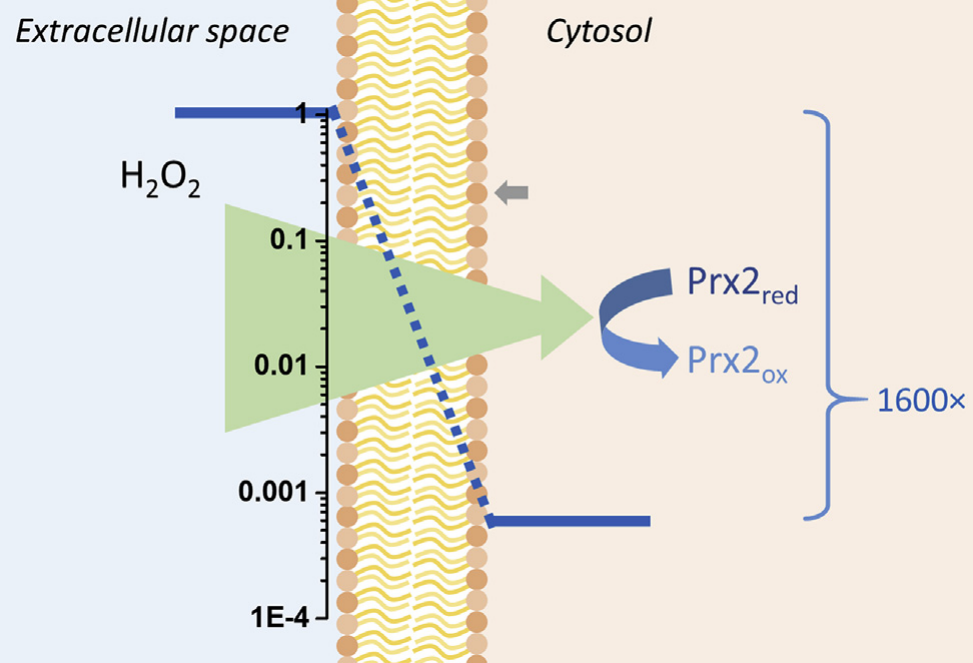
**Concentration gradient of H**_**2**_**O**_**2**_**across the membrane.** Extracellular H_2_O_2_ diffuses across the membrane of the RBC and reacts rapidly with Prx2. The fast intracellular decomposition of H_2_O_2_ (given by the very high rate constant *k* = 1 × 10^8^ M^−1^s^−1^ and the high intracellular concentration of Prx2 in the RBC, 400 μM) and the slightly limited permeability of the membrane to H_2_O_2_ (*P*_m_ = 1.6 × 10^−3^ cm s^−1^) allow the formation of a 1600-fold concentration gradient of H_2_O_2_ across the membrane. In contrast, catalase alone generates a 4.3-fold concentration gradient (*gray arrow*). This large concentration gradient will further promote the net flux of H_2_O_2_ into the RBC, supporting the role of RBCs as efficient sinks of H_2_O_2_ in the vasculature. H_2_O_2_, hydrogen peroxide; Prx2, peroxiredoxin 2; RBC, red blood cell.

Because of experimental limitations, most of our results were obtained under nonphysiological conditions, using either very low hematocrit or very high H_2_O_2_ concentration. However, the mathematical model built using known rate constants for the antioxidant enzymes, validated for a wide range of conditions (9) and now refined with the newly obtained *P*_m_, allows us to explore the otherwise inaccessible physiological conditions of high hematocrit (45%) and low concentration of H_2_O_2_. The estimated half-life for H_2_O_2_ in these conditions is 35.3 ms (Fig. S3). A higher *P*_m_ would allow for even faster rates of clearance of H_2_O_2_ (Fig. S4). In our experimental conditions with high H_2_O_2_ concentration, Prx2 was rapidly oxidized and NADPH depleted (9), and catalase decomposed most H_2_O_2_ (Fig. S1). However, in physiological conditions, Prx2 would remain active, resulting in a much faster decomposition of H_2_O_2_ inside the RBC. As a consequence, a large concentration gradient will be formed across the membrane, estimated to be 1600 times lower in the internal side of the bilayer relative to the external side (Fig. 6). Note that this gradient is much greater than the one formed by catalase alone in the RBC, measured as 4.3 (1/*R*_H2O2_ in Fig. 3). This is because the rate of reaction of Prx2 with H_2_O_2_ is ten times greater and because Prx2 is 20 to 40 times more abundant than catalase (9, 12). The magnitude of the gradient depends inversely on *P*_m_, so that a 10-fold increase in *P*_m_ would lead to a 10-fold decrease in the gradient (Fig. S4).

## Discussion

The partition of H_2_O_2_ in lipid membranes is difficult to determine because H_2_O_2_ is hydrophilic and thus is excluded from the bilayer. Organic solvents have been used for a long time as substitutes for the estimation of the solubility of molecules in the lipid membrane, in particular octanol and hexadecane (25). Using a double-partition approach, we could determine *K*_*D*_° for H_2_O_2_ between water and these solvents and found that H_2_O_2_ is 15 times less soluble in octanol and 122,000 times less soluble in hexadecane than in water at 25 °C. Previous determinations of *K*_*D*_° found that H_2_O_2_ was 14 times less soluble in ether than in water (15), whereas attempts using *n*-heptane were reported to be unsuccessful (27). As a comparison, water is 2.4 million times less soluble in hexadecane than in itself and 25 times less soluble in octanol than in itself (32, 47, 48), indicating that H_2_O_2_ is only slightly less hydrophilic than water. Making simplistic assumptions based on solubility and diffusion, the permeability of membranes composed solely of lipids to H_2_O_2_ was estimated to be *P*_m_ = 7 × 10^−4^ cm s^−1^.

The Gibbs energy profile constructed with the solubility data suggests that the greatest energy barrier to H_2_O_2_ membrane permeation is located at the middle region of the bilayer (Fig. 1). These results agree with and support several molecular dynamics simulations that provide a more detailed free energy distribution across the bilayer (29). The Gibbs energy was estimated to be 33 ± 4 kJ/mol in a DOPC membrane by molecular dynamics simulation (29), in fair agreement with Δ*G*° between hexadecane and water (29.0 kJ/mol; Table 1).

Although there are many studies on the permeability of cell plasma membranes to H_2_O_2_ (reviewed in Ref. (17)), there are only a few that studied the permeability of membranes composed solely of lipids to H_2_O_2_ (15, 27, 28), and none of them provided a value for *P*_m_. An interesting observation from those studies was that an increase in membrane fluidity, caused by temperature, changes from the gel to the fluid liquid crystalline phase, or addition of *n*-nonanol resulted in a significant increase in the permeability to H_2_O_2_ (15, 27).

Herein, we could determine *P*_m_ for different membranes and found that it depended greatly on lipid composition (Table 2). Membranes composed of saturated phospholipids and Chol (DMPC:DPPG:Chol 4:1:5) showed a lower *P*_m_ (4.1 × 10^−4^ cm s^−1^ at 37 °C) than unsaturated phospholipid and Chol membranes (DOPC:POPG:Chol 4:1:5, *P*_m_ = 5.5 × 10^−3^ cm s^−1^ at 37 °C). Because Chol is present at 50%, both membranes are in the liquid ordered state (L_O_), characterized by a higher order at the acyl chains caused by Chol, while retaining free rotation and two-dimensional fluidity (49, 50, 51). However, the condensing effects of Chol are greater on DMPC than on DOPC, evidenced by greater changes in bilayer thickness, apparent area per lipid, and chain order parameters (52). Therefore, membranes containing DOPC:POPG:Chol 4:1:5 will be in a more fluid and less-packed state than the membranes containing the saturated phospholipids, and this would explain the higher *P*_m_. The higher *E*_a_ of permeation through saturated phospholipid membranes is in agreement with a tighter lipid packing than in unsaturated membranes (Table 2).

In membranes of DOPC:Chol 6:4 at 30 °C, *P*_m_ for water was 6.8 × 10^−3^ cm s^−1^ (53). The permeability of membranes to H_2_O_2_ seems to be slightly lower than the permeability to water, probably because of the smaller size of water, a known factor to make molecules more diffusible through membranes than expected based solely on their *K*_*D*_° (25, 54).

The low permeability observed in saturated membranes supports that certain cell membranes may be less permeable to H_2_O_2_ and that channels may be necessary to facilitate the transport of H_2_O_2_. For instance, yeast membranes lacking functional endogenous AQP homologs were shown to be relatively impermeable to H_2_O_2_, and this could be reverted by the expression of some AQPs, such as human AQP8 (18). The involvement of AQP8 in mammalian cell transport of H_2_O_2_ was later confirmed (21, 55). AQP3 was also found to facilitate the transport of H_2_O_2_ in mammalian cells (19, 20). In the case of human AQP1, several studies have indicated that it would not facilitate H_2_O_2_ transport (18, 19), but this position has been challenged and thus remains in debate (23, 24).

The permeability of the human RBC membranes to H_2_O_2_ was found to be 1.0 × 10^−3^ cm s^−1^ and 1.6 × 10^−3^ cm s^−1^, at 25 and 37 °C, respectively. The measured *P*_m_ in RBC indicates that the membrane will limit the diffusion rate of H_2_O_2_ into the cytosol by a factor of 3 × 10^4^ (relative to an equally thick layer of water). However, the very fast reaction of H_2_O_2_ with Prx2 inside the RBC will allow for an efficient and rapid detoxification of H_2_O_2_ (9). A large concentration gradient will be formed across the membrane leading to a net flux of H_2_O_2_ from the extracellular space into the RBC (Fig. 6). As a result, under physiological conditions, very little H_2_O_2_ that diffuses into the RBC will be able to escape, and RBCs will act as efficient sinks of H_2_O_2_ in the vascular space.

In comparison with RBCs from other organisms, *P*_m_ for human RBCs is lower than the one reported for rat RBCs, 1.2 × 10^−2^ cm s^−1^, at 30 °C (15), but similar to the one reported for horse RBCs, 6 × 10^−4^ cm s^−1^, at 20 °C (16). The difference in the permeability of different RBCs to H_2_O_2_ could be caused by differences in membrane lipid composition and/or different membrane protein contribution. Other cellular and organelle membranes show values between 4 × 10^−4^ and 1.6 × 10^−3^ cm s^−1^ at 37 °C (17, 56), thus the human RBC is in the high range but is not much more permeable to H_2_O_2_ than other cells. The permeability of human RBCs is higher than the permeability of DMPC:DPPG:Chol 4:1:5 membranes but lower than the permeability of DOPC:POPG:Chol 4:1:5 membranes (Table 2). The difference in *P*_m_ could occur because the different composition of lipids modifies the permeability of the membrane, the presence of embedded transmembrane proteins in the RBC membrane that modifies the physical properties of the lipids, or because some transmembrane protein facilitated H_2_O_2_ diffusion across the membrane. The *E*_a_ of the permeation process (32 kJ mol^−1^) is lower for RBCs than for liposomes (Table 2), and it is in between the values for diffusional and osmotic permeability of regular and AQP1-null human RBCs to water (19 and 43 kJ mol^−1^, respectively (57)), suggesting the involvement of a transporter protein.

The main suspects in facilitating H_2_O_2_ transport across the RBC membrane were the AQPs. AQP1 is an abundant protein in the membrane of human RBCs. The number of AQP1 in RBC has been reported in 6 to 14 × 10^4^ copies (11, 58). Although it is not an essential protein for cell viability, AQP1 is the most important protein in water transport in the RBCs (57). However, our results on H_2_O_2_ permeability using AQP1-deficient RBCs (Colton-null) showed no difference to normal human RBCs, suggesting that this channel does not facilitate H_2_O_2_ transport in human RBCs.

AQP3 has been repeatedly reported to allow H_2_O_2_ transport across the membrane (19, 20), and this property is probably explained by its larger pore that also transports glycerol. Herein, we did not observe differences between regular and AQP3-deficient human RBCs (GIL-null). The expression level of AQP3 has recently been reported to be 1700 copies per cell, 30 to 100 times less abundant than AQP1 (11). Nonetheless, if H_2_O_2_ transport was exclusively supported by AQP3, as described for glycerol, GIL-null RBCs should have been much less permeable than regular RBCs. Considering that no effect on H_2_O_2_ was observed, AQP3 is not an important route for its transport across RBC membranes.

It cannot be discarded that other proteins may be involved in facilitating H_2_O_2_ transport across the RBC membrane. An interesting candidate is the urea transporter UT-B, which has a unit permeability to water per channel similar to that of AQP1 (44), and is present at 14 to 26 × 10^3^ copies per cell (11).

Considering that the *P*_m_ for H_2_O_2_ across RBC membranes is similar to that of lipid-only liposomes (Table 2); is similar to the membrane osmotic permeability to water in AQP1-null RBCs (*P*_f_ = 2–3 × 10^−3^ cm s^−1^ at 26 and 20 °C) (57, 59); is similar in regular, AQP1-null, and AQP3-null cells; does not show saturation; strongly suggests that the main mechanism of H_2_O_2_ permeation across RBC membranes is simple diffusion across the lipid fraction.

Although in some other cell types, specific AQPs have been shown to facilitate the diffusion of H_2_O_2_ (peroxiporins), our results suggest that AQPs are not the only possible routes for H_2_O_2_ diffusion into cells. Depending on the cell type (and likely organelle), the lipid fraction of the membrane may be sufficiently permeable to H_2_O_2_, or still unidentified membrane proteins may be involved in facilitating H_2_O_2_ transport across cellular membranes.

## Conclusions

In summary, the present study revealed several aspects of H_2_O_2_ interaction with membranes: (1) the solubility of H_2_O_2_ in the solvents octanol and hexadecane, which resemble the membrane interior, is 15 and 122,000 times lower than in water, confirming that H_2_O_2_ will face a large thermodynamic barrier when diffusing across lipid membranes; (2) the permeability of phospholipid:Chol liposome membranes to H_2_O_2_ depends on the composition of the lipids and increases with acyl-chain unsaturation; (3) the permeability of RBC at 37 °C is 1.6 × 10^−3^ cm s^−1^, which is 3 × 10^4^ times lower than an equally thick layer of water; (4) the fast reaction of H_2_O_2_ with cytosolic Prx2 results in the formation of a 1600-fold concentration gradient of H_2_O_2_ across the membrane; (5) the estimated half-life of H_2_O_2_ in blood under physiological conditions is 34.5 ms; and (6) AQPs are not involved in facilitating H_2_O_2_ diffusion across the membrane of RBCs, and diffusion occurs likely through the lipid fraction or through a still unidentified membrane protein.

## Experimental procedures

### Materials

Chemical reagents were obtained from Sigma, Applichem, and Acros Organics. Lipids were obtained from Avanti Polar Lipids and Larodan. Work solutions of H_2_O_2_ were prepared daily and quantified by spectrophotometry, ε(240 nm) = 39.4 M^−1^ cm^−1^ (60).

The studies using human blood were conducted in accordance with the Declaration of Helsinki. Blood was obtained from volunteer donors after informed consent at the Cátedra y Departamento de Medicina Transfusional del Hospital de Clínicas, Facultad de Medicina, Universidad de la República, Montevideo, Uruguay. The research protocol was approved by the Hospital's Ethics Committee. Packed RBCs were obtained by standard techniques without leukoreduction, as described before (61). For AQP1-deficient and AQP3-deficient RBC experiments, control, Colton-null, and GIL-null RBCs were obtained from cryopreserved samples from the Rare Blood Collection at the Centre National de Référence pour les Groupes Sanguins de l’Institut National de la Transfusion Sanguine, Paris, France. Spectrophotometric measurements were performed in a Varian Cary 50 (Agilent) spectrophotometer in wildtype experiments. In AQP1-defecient and AQP3-deficient RBC experiments, a NanoDrop 2000c spectrophotometer (Thermo Fisher Scientific) was used for simple spectrophotometric reads, and an SFM400 Stopped Flow (Bio-Logic) was used for continuous absorbance measurements.

### H_2_O_2_ partition experiments

The solubility of H_2_O_2_ in organic solvents is very low because H_2_O_2_ is hydrophilic. Furthermore, most methods for H_2_O_2_ quantification are based on water. Therefore, we devised a double-partition method involving a first step of equilibration of H_2_O_2_ between water (w1) and organic solvent (os1), careful extraction of the organic phase to prevent carrying any water, and then a second step where this organic phase (os2) was equilibrated with new water (w2). Every volume was accurately determined (V_os1_, V_w1_, V_os2_, and V_w2_). Note that V_os2_ was approximately half that of V_os1_ to avoid any contaminating water. The water used was saturated with the organic solvent of interest, and the organic solvents were saturated with water to prevent artificial increases in the solubility of H_2_O_2_. The equilibration of a known amount of H_2_O_2_ proceeded at controlled temperature in a water bath with frequent mixing for 30 min. The phases were then separated by centrifugation, and a fraction of the organic solvent (on top) was removed and placed in a new container with water, and the equilibration was repeated. The concentration of H_2_O_2_ was quantified both in the initial and final water phases.

The following rationale was used to calculate the *K*_*D*_° of H_2_O_2_ between the organic phase and water. First, *K*_*D*_° is the same in both steps, while the concentrations in each phase change.

(3) $$K_{D}\text{° }=\;{\left[{\text{H}}_{2}{\text{O}}_{2}\right]\text{os}}_{1}/{\left[{\text{H}}_{2}{\text{O}}_{2}\right]\text{w}}_{1}$$

(4) $$K_{D}\text{° }=\;{\left[{\text{H}}_{2}{\text{O}}_{2}\right]\text{os}}_{2}/{\left[{\text{H}}_{2}{\text{O}}_{2}\right]\text{w}}_{2}$$

Because of mass conservation, the total number of moles in the second equilibrium (n_T2_) is:

(5) $${\text{n}}_{\text{T}2}={\left[{\text{H}}_{2}{\text{O}}_{2}\right]}_{{\text{w}}_{2}}{\text{V}}_{\text{w}2}+{\left[{\text{H}}_{2}{\text{O}}_{2}\right]\text{os}}_{2}{\text{V}}_{\text{os}2}$$

that can be expressed as

(6) $${\text{n}}_{\text{T}2}={\left[{\text{H}}_{2}{\text{O}}_{2}\right]\text{w}}_{2}\left({\text{V}}_{\text{w}2}+K_{D}{\text{°V}}_{\text{os}2}\right)$$

With this information, we can calculate the concentration in the organic solvent in the first equilibrium:

(7) $${\left[{\text{H}}_{2}{\text{O}}_{2}\right]\text{os}}_{1}={\text{n}}_{\text{T}2}/{\text{V}}_{\text{os}2}$$

So that we can replace in Equation 3 and rearrange to obtain:

(8) $$K_{D}\text{°}={\left[{\text{H}}_{2}{\text{O}}_{2}\right]\text{w}}_{2}{\text{V}}_{\text{w}2}/\left(\left({\left[{\text{H}}_{2}{\text{O}}_{2}\right]\text{w}}_{1}-{\left[{\text{H}}_{2}{\text{O}}_{2}\right]\text{w}}_{2}\right){\text{V}}_{\text{os}2}\right)\;$$

The quantification of H_2_O_2_ was done using horseradish peroxidase (0.05 U/ml) and 2,2′-azino-*bis*(3-ethylbenzothiazoline-6-sulphonic acid) (0.1 mM) in 0.1 M potassium phosphate buffer at pH 5, measuring the 2,2′-azino-*bis*(3-ethylbenzothiazoline-6-sulphonic acid) radical cation at 734 nm by spectrophotometry (62). Calibration curves were constructed using H_2_O_2_ quantified at 240 nm (see aforementioned). The response was linear up to 40 μM. Different conditions were assayed until optimal initial concentrations in w1 for octanol and hexadecane were found to be 5 mM and 2 M H_2_O_2_, respectively.

### Thermodynamics of partition

The Gibbs energy of partition (Δ*G*°), indicating the energy required for the transfer of H_2_O_2_ from water to the organic solvent, was calculated from *K*_*D*_°:

(9) $$\Delta G\text{°}=-RT\text{ ln}\left(K_{D}\text{°}\right)$$

where *R* is the universal gas constant (8.314 J mol^−1^ K^−1^) and *T* is the absolute temperature.

The enthalpy and entropy of the partition (Δ*H*° and Δ*S*°) were calculated from *K*_*D*_° at 25 and 37 °C, using the van't Hoff equation:

(10) $$\text{ln}\left(K_{D}^{\text{ o}}\right)=-\frac{H{}^{\circ}}{R}\frac{1}{T}+\frac{S{}^{\circ}}{R}$$

### Preparation of catalase-encapsulated liposomes

The desired final composition of the membrane was made by mixing chloroform solutions of the lipids and then drying by nitrogen stream and then vacuum for 2 h. Typical preparations contained 10 mg of lipids and were composed of either DMPC:DPPG:Chol (4:1:5 mole fraction) or DOPC:POPG:Chol (4:1:5 mole fraction).

The dried lipids were then incubated with a work solution containing 2 mg/ml catalase in 50 mM sodium phosphate at pH 7 buffer. Catalase solution was previously filtered through 0.22 μm pore filters (polyvinylidene fluoride; Millipore). The lipids were allowed to hydrate in this solution for 30 min with frequent mixing. Liposomes were then prepared by extrusion through 200 nm or 1000 nm nucleopore membranes (Whatman) 15 times using a syringe extruder (Avanti). To separate the catalase-encapsulated liposomes from free catalase, a Superdex 200 (10/300 GL; GE Healthcare) column coupled to an HPLC (Agilent; 1260), using 50 mM sodium phosphate at pH 7 at 0.5 ml/min as the mobile phase, was used. Because of their large size, liposomes elute very early (17 min), whereas catalase is retarded and elutes later (30 min). Liposomes were collected and used for permeability experiments. The size of the liposomes was consistent with the nominal pore value of the nucleopore filter membranes to within 5%, as confirmed by dynamic light scattering (Brookhaven Instruments).

### Determination of permeability coefficients of lipid membranes

The calculation of the *P*_m_ is based on the concept of enzyme latency (41). In some cases, enzymes encapsulated by membranes show a lower activity than enzymes free in solution, particularly if the membrane is partially permeable to the substrate. The diffusion of the substrate is slowed, and a concentration gradient forms across the membrane that is observed as a different enzyme activity. In steady state, the ratio of membrane-encapsulated and free enzyme activity (*R*_H2O2_) will yield the concentration gradient. For H_2_O_2_, the catalase activity is measured by following the rate of H_2_O_2_ (10 mM) decomposition by spectrophotometry at 240 nm, by liposome-encapsulated and disrupted liposome catalase (16, 41). Liposomes containing catalase were disrupted by filtration through 30 nm nucleopore filters (Whatman) 11 times in a syringe mini extruder (Avanti). Complete equilibration of catalase between external and internal volumes was confirmed by measuring catalase activity at different cycles of extrusion, which showed that seven cycles already yielded maximal catalase activity. Initial rates of H_2_O_2_ decomposition were obtained from linear regression and plotted as a function of liposome volume. The slopes of these secondary plots (named *k*_lipo_ and *k*_dis_ for intact and disrupted liposomes, respectively) were used to calculate *R*_H2O2_ (*k*_lipo_/*k*_dis_) and then *P*_m_ according to Equation 2. *k*_*catalase*_ was determined by measuring the rate of decomposition of H_2_O_2_ by different dilutions of the catalase work solution and then extrapolated to the concentration in the work solution. This *k*_*catalase*_ was determined individually for each liposome preparation.

### Estimation of activation energy for H_2_O_2_ permeation

*P*_m_ was determined at different temperatures between 10 and 40 °C following the previously described protocol. The obtained values were used to construct a ln(*P*_m_) *versus T*^−1^ plot, from which the *E*_a_ was calculated as shown in Equation 11:

(11) $$\text{ln}\left(P_{\text{m}}\right)=\ln\left(\text{A}\right)-\frac{E_{\text{a}}}{R}\left(\frac{1}{\text{T}}\right)$$

where A is the pre-exponential factor and *R* is the universal gas constant. The *E*_a_ for catalase is very low (2.5 kJ/mol, (63)), and the *E*_a_ for *P*_m_ was not corrected for its contribution.

### RBC preparation

Before each experiment, the RBCs were washed three times in Hank's balanced salt solution (137 mM NaCl, 5.4 mM KCl, 0.25 mM Na_2_HPO_4_, 1 g l^−1^ glucose, 0.44 mM KH_2_PO_4_, 1.3 mM CaCl_2_, 1.0 mM MgSO_4_ and 4.2 mM NaHCO_3_, and pH 7.4) by centrifugations at 900*g* for 4 min at room temperature. After that, the cells were resuspended in the same solution and diluted until the hematocrit was approximately 0.06%. The experiments with RBCs were performed using freshly obtained blood, on the same day of the experiment. The experiments with RBCs lacking AQP1 or AQP3 were performed after recovering the frozen samples and compared with cryopreserved normal RBCs.

### Determination of H_2_O_2_ permeability coefficient of RBCs

Suspensions of intact RBCs with increasing hematocrits, obtained *via* dilution of the stock, were mixed with 10 mM H_2_O_2_ in Hank's balanced salt solution. H_2_O_2_ consumption was then measured spectrophotometrically at 240 nm for 1 min at 37 °C. The same procedure was performed using lysed cells, generated by freezing part of the original stock of RBCs. Initial rates were obtained from linear regression and plotted as a function of HbO_2_ concentration, determined through absorbance measurements at 577 nm (ε(HbO_2_) = 15 mM^−1^ cm^−1^, (64)). The slopes of these secondary plots (named *k*_RBC_ and *k*_lys_ for intact and lysed RBCs, respectively) were used to calculate *R*_H2O2_ and then *P*_m_ following Equation 10 (41). Hemolysis was very low in these experiments. Free hemoglobin after treatment with H_2_O_2_ was the same as the control with buffer and less than 2% of the equivalent lysed RBCs. Considering that intact RBCs had 23% of the catalase activity compared with lysed RBCs, more than ten times than that expected from the contribution of hemolysis, it can be ascertained that in intact RBC experiments, most of the catalase activities were derived from RBC-encapsulated catalase. Because some of the hemolysis may also occur during the centrifugation to sediment RBCs, no corrections for hemolysis were introduced.

In the case of RBCs, *k*_*catalase*_ is the pseudo–first-order constant for H_2_O_2_ removal by catalase inside the RBC (determined by extrapolation of the *k*_lys_ value for a concentration of 20 mM HbO_2_), *R*_H2O2_ represents the *k*_RBC_/*k*_lys_ ratio, A is the surface area (1.4 × 10^−6^ cm^2^) and V the volume (9 × 10^−11^ cm^3^) of the RBC (65, 66). Determinations of *P*_m_ were done using three biological replicates, each measured three separate times (n = 9) in order to determine *P*_m_ in basal conditions (37 °C, pH 7.4). Mathematical simulations showed that under these experimental conditions, catalase was effectively the most important enzymatic system decomposing H_2_O_2_ (>97%; Fig. S1).

### Water permeability and inhibition of AQP1

RBC suspensions at 0.5% hematocrit were incubated with 0.5 mM HgCl_2_ or pCMBS in PBS (137 mM NaCl, 2.7 mM KCl, 8 mM Na_2_HPO_4_, and 2 mM KH_2_PO_4_) for 30 min at room temperature. Inhibition of water passage through AQP1 by these compounds was checked using an SX Stopped-Flow Spectrometer (Applied Photophysics) to follow changes in RBC volume after mixing them with an equal volume of a hypertonic solution of 250 mM sucrose at 10 °C (67). Water efflux was recorded by 90° light scattering measurements of 2 s of duration, and the resulting time courses were fitted to an exponential model. The observed constants (*k*_water_) were then contrasted with those obtained in assays involving untreated RBCs. The effect of HgCl_2_ and pCMBS in H_2_O_2_ metabolization was evaluated by taking a 40 μl aliquot of the 0.5% hematocrit stock, treated or untreated with each inhibitor, and adding 10 mM H_2_O_2_ in a final volume of 1 ml. H_2_O_2_ removal was monitored by absorbance measurements at 240 nm, allowing the calculation of initial rates of decomposition.

### Glycerol permeability and inhibition of AQP3

Phloretin was tested and used as an AQP3 inhibitor. RBC suspensions of 0.5% hematocrit were incubated in hypotonic 0.7× PBS for 30 min at room temperature. Afterward, a fraction of the sample was treated with 0.5 mM phloretin for another 30 min, whereas the rest remained untreated. Control or treated RBCs were then mixed in equal volumes with a solution of 100 mM glycerol in hypotonic buffer using an SX Stopped-Flow Spectrometer (Applied Photophysics) (68). Changes in cell volume corresponding to the efflux of water and subsequent influx of glycerol were followed by light scattering measurements for 40 s. The resulting time courses were fitted to a double exponential function with two observed constants corresponding to the ascending and descending phases, the latter (*k*_glycerol_) being used to evaluate the effect of phloretin in glycerol transport (68). To evaluate the effect in H_2_O_2_ consumption, the cells (0.5% hematocrit) were incubated with 0.5 mM phloretin in 1× PBS. The reaction with H_2_O_2_ was studied in the same conditions as described previously.

### Statistical analysis

Data were analyzed using GraphPad Prism 6 (GraphPad Software, Inc). Statistical analyses were performed by one-way ANOVA and Dunnett's post hoc test to perform multiple comparison tests. Differences with *p* < 0.05 were considered statistically significant.

## Data availability

All data are contained within the article. Copasi files are to be shared upon request to Matías Möller, mmoller@fcien.edu.uy.

## Supporting information

This article contains supporting information (9, 16, 17, 41, 42, 61, 69, 70, 71, 72).

## Conflict of interest

The authors declare that they have no conflicts of interest with the contents of this article.
