# Supplementary material for: The permeability of human red blood cell membranes to hydrogen peroxide is independent of aquaporins
Source: J Biol Chem. 2021 Dec 18;298(1):101503. doi: 10.1016/j.jbc.2021.101503 (PMC8753180; doi:10.1016/j.jbc.2021.101503)
Supplement: Supplemental Figures S1–S4 [file mmc1.pdf]

Supporting information

The permeability of human red blood cell membranes to hydrogen peroxide is independent of aquaporins

Florencia Orrico [a,b,c,&], Ana C. Lopez [a,b,c,&], Daniela Saliwonczyk [b,d], Cecilia Acosta [b,d], Ismael Rodriguez-Grecco [d], Isabelle Mouro-Chanteloup [e,f], Mariano Ostuni [e,f], Ana Denicola [a,c], Leonor Thomson [b,c] *, Matias N. Möller [a,c] *

[a] Laboratorio de Fisicoquímica Biológica, Instituto de Química Biológica, Facultad de Ciencias, Universidad de la República, Montevideo 11400, Uruguay

[b] Laboratorio de Enzimología, Instituto de Química Biológica, Facultad de Ciencias, Universidad de la República, Montevideo 11400, Uruguay

[c] Centro de Investigaciones Biomédicas (CEINBIO), Universidad de la República, Montevideo 11800, Uruguay

[d] Departamento de Medicina Transfusional, Hospital de Clínicas, Facultad de Medicina, Universidad de la República.

[e] Université de Paris, UMR_S1134, BIGR, Inserm, F-75015 Paris, France

[f] Laboratoire d'Excellence GR-Ex, Paris, France

[&] Both authors contributed equally.

* Corresponding authors: Laboratorio de Fisicoquímica Biológica, Instituto de Química Biológica, Facultad de Ciencias, Iguá 4225, Montevideo 11400, Uruguay. Tel/fax: (+598) 2525 0749. E-mail: mmoller@fcien.edu.uy; Laboratorio de Enzimología, Instituto de Química Biológica, Facultad de Ciencias, Iguá 4225, Montevideo 11400, Uruguay. Tel/fax: (+598) 2525 0749. E-mail: lthomson@fcien.edu.uy

Enzyme latency method used to determine P_m

The advantage of the latency method is that the rate of the enzymatic reaction does not have to be much higher than the diffusion rate across the membrane, but only slightly higher or in the order. It considers that H_2O_2 can diffuse into the vesicle and react with catalase and part of it diffuse back to the external solution. A steady-state is achieved rapidly and then a competition between catalase-decomposition and diffusion ensues, leading to the formation of a medium-low concentration gradient of H_2O_2 across the membrane. The slow consumption of H_2O_2 allows to measure H_2O_2 disappearance for relatively long times (1 minute to be within initial rate conditions, less than 10% of initial H_2O_2 consumed). The gradient formed by catalase at the conditions used was estimated to be around 4.

This method was used previously by Nicholls (1) and Antunes (2,3). Others have used a very similar approach to determine either P_m or the gradient of concentrations of H_2O_2 across cellular membranes (reviewed by Möller (4)). A detailed explanation is given below.

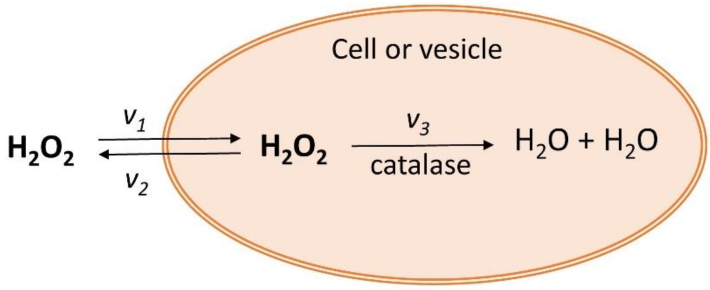

$v_1 = k_{diff}[H_2O_2]_{out}$ $\quad k_{diff} = P_m \cdot A / V$

$v_2 = k_{diff}[H_2O_2]_{in}$

$v_3 = k_{cat}[cat][H_2O_2]_{in} = k_{catalase}[H_2O_2]_{in}$

At steady-state conditions:

$v_1 = v_2 + v_3$

$k_{diff}[H_2O_2]_{out} = (k_{diff} + k_{catalase})\,[H_2O_2]_{in}$

Scheme S1. Modified from Antunes to account only for catalase (2).

The pseudo-first order rate constant of H_2O_2 consumption by catalase (k_{cat}[cat] = $k_{catalase} \sim 40\ s^{-1}$) inside the liposome is very similar to the first order constant associated to H_2O_2 diffusion across the membrane ($k_{diff} \sim 20\ s^{-1}$)(Scheme S1). A large fraction of H_2O_2 that diffuses into the liposome will react with catalase and some will diffuse back to the extravesicular space. As a result, a medium-low gradient of H_2O_2 concentration will form across the membrane.

It follows from the last equation in Scheme S1 that

$[H_2O_2]_{in} / [H_2O_2]_{out} = k_{diff} / (k_{diff} + k_{catalase})$

R_{H2O2} is defined as:

$R_{H2O2} = [H_2O_2]_{in} / [H_2O_2]_{out}$

So R_{H2O2} is also:

$R_{H2O2} = k_{diff} / (k_{diff} + k_{catalase})$

Then

$R_{H2O2} (k_{diff} + k_{catalase}) = k_{diff}$

$R_{H2O2}\, k_{catalase} = k_{diff} (1- R_{H2O2})$

Since

$k_{diff} = P_m A/V$

then

$P_m = k_{catalase} R_{H2O2} / ((1- R_{H2O2}) A/V)$ (Eq. 2 in text)

R_{H2O2} can be experimentally obtained from the ratio of reaction rates in whole liposomes vs disrupted liposomes with no permeability barrier:

$k_{lipo} = [H_2O_2]_{in}\ k_{catalase}$ / (liposome suspension volume)

$k_{dis} = [H_2O_2]_{out}\ k_{catalase}$ / (lysed liposome suspension volume)

The experiments were done so that (lysed liposome suspension volume) corresponds to the same initial (liposome suspension volume), so both have the same concentration of catalase, and $k_{catalase}$ is the same for both, thus,

$R_{H2O2} = k_{lipo} / k_{dis} = [H_2O_2]_{in} / [H_2O_2]_{out}$

The important assumption that was made was that the concentration of catalase inside liposomes is the same as the concentration of catalase in the *catalase work solution* that was used to make them. That *catalase work solution* was used to determine $k_{catalase}$ and use it in Eq. 2. It was a reasonable assumption because the liposomes were prepared and used them the same day, and they were found to be stable (same catalase activity) for at least three days. Furthermore, the resulting P_m values were found to be consistent between batches from different preparations.

Mathematical modeling of H_2O_2 consumption by antioxidant systems in RBC

The mathematical modeling of reactions was based on the previous model (5) built in Copasi (6). The reactions related to H_2O_2 reduction by GSH and by GPx were corrected relative to the previous model. The modification of these reaction did not modify previous findings, but the model was updated. The model was used to simulate H_2O_2 consumption by red blood cells and determine the relative contribution of the different antioxidant enzymes and also the effect of different membrane permeability coefficients (P_m) on H_2O_2 consumption rates (Figure S1A and B, respectively).

Modifications introduced to previous model

Glutathione oxidation by H_2O_2

H2O2{Intracel} + GSH -> GSOH + Q_GSH1

K = 0.89 $M^{-1}s^{-1}$ (7)

Q terms are artificial insertions (dummies) to quantify H_2O_2 consumption by that particular route.

GSOH + GSH -> GSSG + Q_GSH2

k = 1 × 10^5 $M^{-1}s^{-1}$ (8)

H_2O_2 reduction by GPx

H2O2{Intracel} + GPx -> GPxOH + Q_Gpx

k = 4.1 × 10^7 $M^{-1}s^{-1}$ (9)

GPxOH + GSH -> GPx-SG

k = 7.3 × 10^5 $M^{-1}s^{-1}$ (9)

GPx-SG + GSH -> GPx + GSSG

k = 1 × 10^8 $M^{-1}s^{-1}$ (assumed)

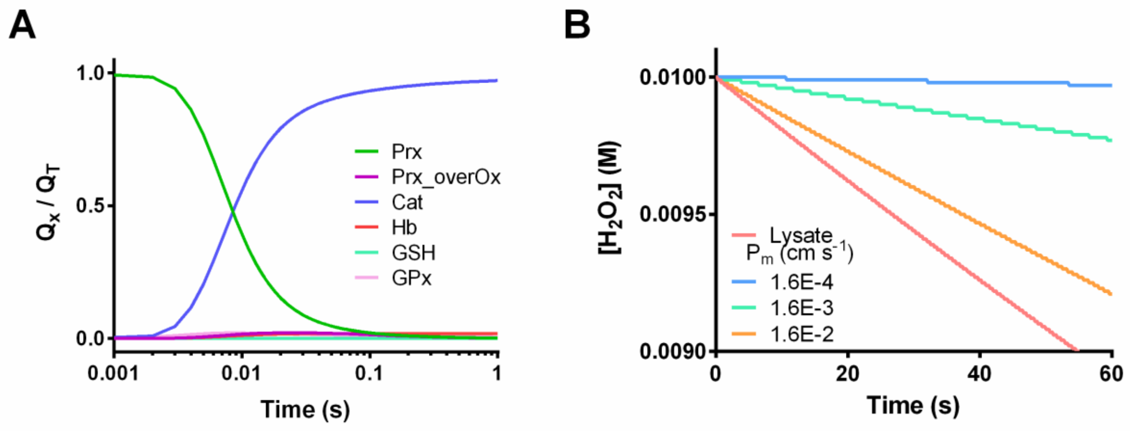

Figure S1. Kinetic simulations of H_2O_2 consumption. A. At 0.2% Hct, Prx2 is the main enzyme responsible for H_2O_2 decomposition until all Prx2 is oxidized and NADPH is depleted (See (5)). After that (approx. 0.01s), catalase is the main enzyme responsible for H_2O_2 decomposition, accounting for more than 97% after 1 s. The experimental conditions used in this work will therefore probe catalase activity in RBC. **B.** The rate of H_2O_2 consumption was simulated at 0.02% Hct using different permeability coefficients, namely: 1.6×10^{-4} cm s^{-1}, 1.6×10^{-3}, 1.6×10^{-2} cm s^{-1}. The lysate was simulated considering P_m = 120 cm s^{-1}. For modeling purposes, the $k_{diffusion} = P_m$. Area/Volume were 2.4, 24, 240, and 1.9×10^6 s^{-1}. It is evident that the rate of H_2O_2 added to a suspension of RBC will be consumed at different rates depending on the P_m.

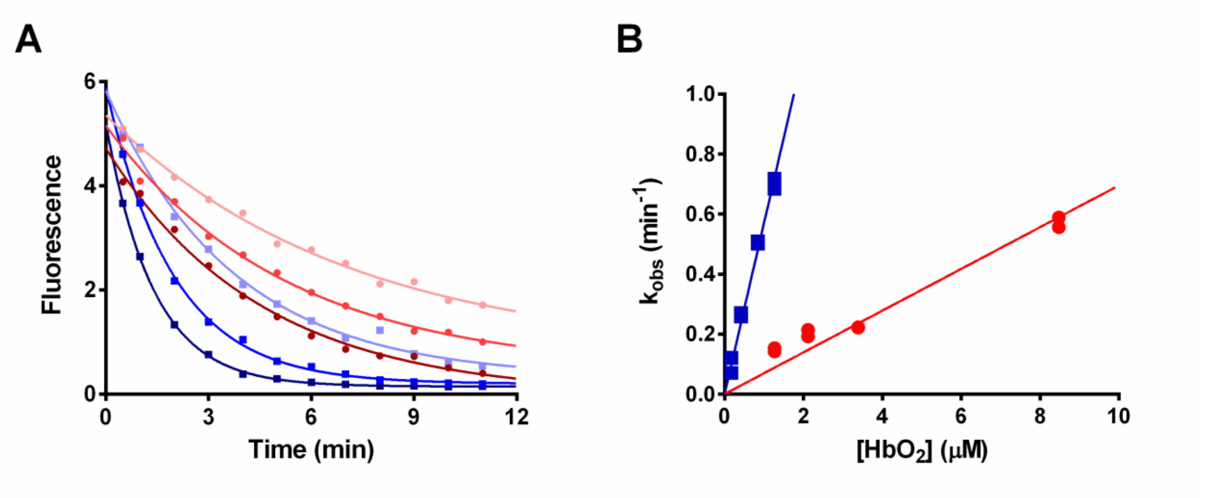

Figure S2. **Determination of H_2O_2 permeability of RBC by pHPA-HRP method.** Suspensions of intact and disrupted cells with different HbO_2 concentrations were mixed with 50 µM H_2O_2 in HBSS solution at 37°C, pH 7.4. H_2O_2 consumption was tracked by taking a 100 µl aliquot every 60 seconds and incubating it in a 96 well plate (Greiner Bio-One GmbH, Germany) with 100 µl of stopping solution containing 10 mM p-hydroxyphenylacetic acid (pHPA) and 0.1 g L^{-1} horseradish peroxidase (HRP). In this conditions, HRP is activated by the reaction with the remaining H_2O_2 in the mix and oxidizes pHPA forming fluorescent dimers (λ_{ex}=325 nm, λ_{em}=400 nm) (10). Fluorescence measurements were carried out using a plate reader Varioskan Flash (Thermo, Finland). **A.** Time courses obtained for intact (1.3 to 8.5 µM HbO_2, red tones) and disrupted RBC (0.17 to 1.3 µM, blue tones). Pseudo-first order constants (k_{obs}) for H_2O_2 removal were obtained by exponential regression. **B.** Secondary plot for permeability determination. The ratio between the slopes k_{RBC} (red) and k_{lys} (blue) was used to calculate the permeability coefficient, $P_m = 1.7 \times 10^{-3}$ cm s^{-1}.

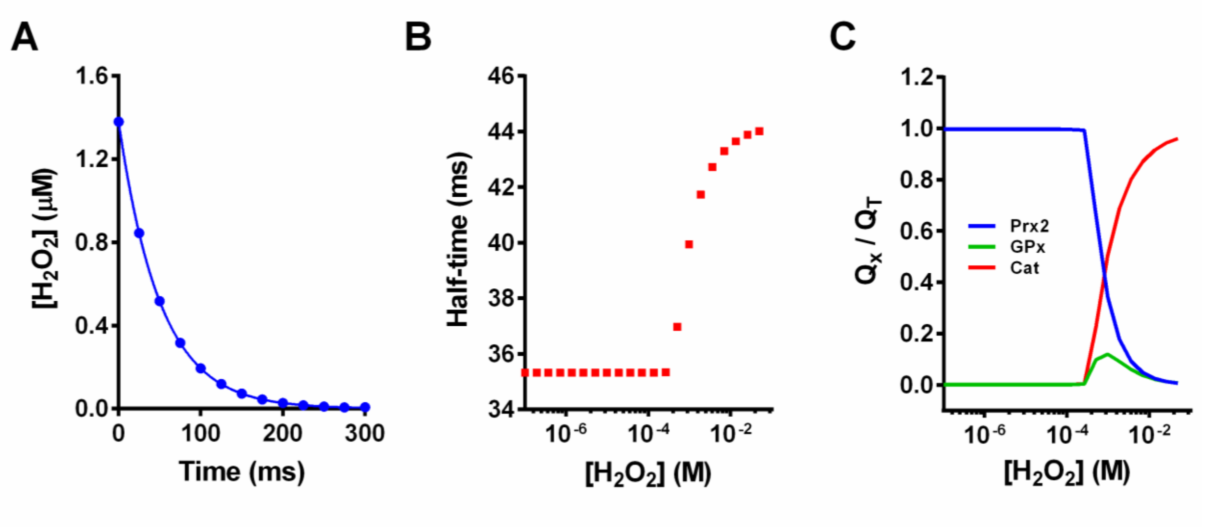

Figure S3. Kinetics of H_2O_2 clearance by RBC in physiological conditions. Using the mathematical model described above, we determined the rate of clearance of extracellular H_2O_2 by a physiological density of RBC, equivalent to 45% hematocrit. **A)** The clearance rate could be exactly described by an exponential decay, from which a rate constant *k* was obtained. To facilitate the visualization, the half-life corresponding to this k was calculated: $t_{0.5} = \text{Ln}(2)/k$. The half-life for 1.4 µM H_2O_2 was 35.2 ms. **B)** The half-life of H_2O_2 was calculated for different initial concentrations of H_2O_2. It was found that it was constant at 35.3 ms up to 300 µM H_2O_2. Above that concentration the half-life increased to 44 ms. **C)** Looking into the relative contribution of the different antioxidant systems of the RBC in clearing H_2O_2 Q_x/Q_T, it can be seen that at low concentrations of H_2O_2, virtually all of it will be consumed by Prx2. Above 300 µM H_2O_2, Prx2 is completely oxidized and cannot be reduced because Trx is also oxidized and NADPH reserves are depleted (5). Above 1 mM H_2O_2, catalase is the main enzyme responsible for H_2O_2 clearance. The lower rate of reaction and lower concentration than Prx2 lead to the increase in H_2O_2 half-life observed in B.

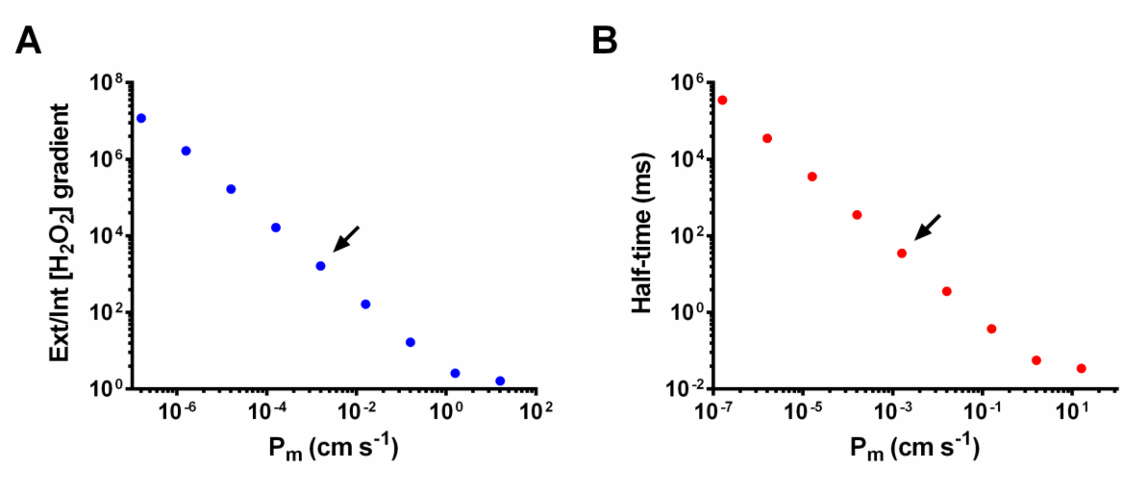

Figure S4. Effects of modifying the permeability of the membrane to H_2O_2 in the concentration gradient and the half-life of H_2O_2. The model included 45% hematocrit and an initial extracellular concentration of H_2O_2 of 10 µM. **A)** The concentration gradient of H_2O_2 across the membrane was calculated form the external and internal concentration of H_2O_2 at initial times of the reaction. The gradient responds inversely to P_m, increasing at lower P_m and decreasing at higher P_m. The black arrow indicates the normal conditions. The proportionality holds for a wide range of P_m values, and only breaks in conditions where diffusion across the membrane approaches the value of diffusion in water. **B)** The half-life of H_2O_2 also responds inversely to P_m. Increasing P_m leads to a lower half-life.

References

1. Nicholls, P. (1965) Activity of catalase in the red cell. *Biochimica et Biophysica Acta (BBA)-Enzymology and Biological Oxidation* **99**, 286-297
2. Antunes, F., and Cadenas, E. (2000) Estimation of H 2 O 2 gradients across biomembranes. *FEBS letters* **475**, 121-126
3. Marinho, H. S., Cyrne, L., Cadenas, E., and Antunes, F. (2013) The cellular steady-state of H2O2: latency concepts and gradients. *Methods in enzymology* **527**, 3-19
4. Möller, M. N., Cuevasanta, E., Orrico, F., Lopez, A. C., Thomson, L., and Denicola, A. (2019) Diffusion and transport of reactive species across cell membranes. in *Bioactive Lipids in Health and Disease*, Springer. pp 3-19
5. Orrico, F., Möller, M. N., Cassina, A., Denicola, A., and Thomson, L. (2018) Kinetic and stoichiometric constraints determine the pathway of H2O2 consumption by red blood cells. *Free Radical Biology and Medicine* **121**, 231-239
6. Hoops, S., Sahle, S., Gauges, R., Lee, C., Pahle, J., Simus, N., Singhal, M., Xu, L., Mendes, P., and Kummer, U. (2006) COPASI—a complex pathway simulator. *Bioinformatics* **22**, 3067-3074
7. Winterbourn, C. C. (2013) The biological chemistry of hydrogen peroxide. in *Methods in enzymology*, Elsevier. pp 3-25
8. Trujillo, M., Alvarez, B., and Radi, R. (2016) One-and two-electron oxidation of thiols: mechanisms, kinetics and biological fates. *Free radical research* **50**, 150-171
9. Takebe, G., Yarimizu, J., Saito, Y., Hayashi, T., Nakamura, H., Yodoi, J., Nagasawa, S., and Takahashi, K. (2002) A comparative study on the hydroperoxide and thiol specificity of the glutathione peroxidase family and selenoprotein P. *Journal of Biological Chemistry* **277**, 41254-41258
10. Amen, F., Machin, A., Touriño, C., Rodríguez, I., Denicola, A., and Thomson, L. (2017) N-acetylcysteine improves the quality of red blood cells stored for transfusion. *Archives of Biochemistry and Biophysics* **621**, 31-37
